# Probable Enalapril Hepatotoxicity in a 13-Year-Old Male Western Lowland Gorilla: Pharmacologic Risks and Vigilance

**DOI:** 10.3389/fvets.2019.00353

**Published:** 2019-10-11

**Authors:** Kathryn C. Gamble, Jessica N. Lovstad, Kate A. Gustavsen

**Affiliations:** Lincoln Park Zoo, Veterinary, Chicago, IL, United States

**Keywords:** angiotensin converting enzyme inhibitors, adverse drug reaction, browse, liver, pharmaceutical

## Abstract

A 13-year-old male Western lowland gorilla presented acutely with a precipitous decline in health status from liver disease. Through diagnostic assessment, including serum chemistries and advanced imaging, it was diagnosed with probable hepatotoxicity resulting from its prescribed medication, enalapril. As one of several angiotensin converting enzyme inhibitors (ACE-I) available to zoo veterinarians, enalapril had been administered for treatment of mild ventricular hypertrophy diagnosed during routine examination 2.5 years prior to the presentation. The gorilla made a complete recovery with discontinuation of this medication, and provision of hepatoprotectants. Hepatotoxicity has been documented in humans receiving this product as an adverse drug reaction and is considered both rare and unpredictable in occurrence. In this event, an association was suspected with indulgent consumption of mulberry browse (*Morus* sp.) offered as nutritional enrichment immediately prior to clinical presentation and had potential impact on hepatic cytochrome P450 metabolism of the enalapril. Although ACE-I are important medications in this taxon due to its predisposition to cardiac disease, this event underscores the need for vigilance on the part of veterinarians and managers whenever pharmaceuticals are administered. Most drugs are modeled in a limited number of species but utilized in a wide variety, and unintended results are possible.

## Introduction

### Case Presentation

An 10.5-years-old male Western lowland gorilla (*Gorilla gorilla gorilla*) was anesthetized with tiletamine-zolazepam (Telazol, Zoetis, Inc., Kalamazoo, Michigan, 500 mg, i.m. via dart) and isoflurane (Isothesia, Henry Schein Animal Health, Dublin, Ohio) in oxygen for routine preventive medical assessment. Although generally healthy, it was diagnosed with mild bilateral atrial enlargement, and borderline biventricular hypertrophy by consultation with a cardiologist physician. Early intervention was recommended as this gorilla's sire had been diagnosed with progressive cardiac disease, and due to the known species tendency for cardiac insufficiency. Treatment was initiated with a conservative dose of enalapril (Wockhardt Ltd., Parsippany, New Jersey 07054, USA; 2.5 mg PO, SID). The gorilla was confirmed as clinically healthy and without progression of cardiac disease during a similar preventive medical assessment after 2 years of enalapril treatment.

Approaching 3.5 years of treatment, the now 13-years-old gorilla presented acutely with anorexia, apparent gastrointestinal discomfort, and malaise. The only presenting history of note was that this individual and its three co-housed, nearly age-matched bachelor conspecifics had received mulberry (*Morus* sp.) as browse on the preceding day, which had been consumed extensively to a degree considered atypical for this troop. The gorilla accepted an oral gastroprotectant (famoditine, Teva Pharmaceuticals USA, Inc., North Wales, Pennsylvania) for 4 days, and remained compliant with enalapril.

The severity of clinical signs progressed markedly over 4 days. On the fifth day, the gorilla was anesthetized as previously described to complete a diagnostic assessment. At this examination, the gorilla was found to be moderately dehydrated and febrile, but no specific system of concern was identified by physical examination or intraprocedural clinical pathology. Cardiology consultation was repeated and confirmed no progression of the underlying cardiac condition. However, during echocardiography, the liver was noted to be mildly hyperechoic focally at the portal areas as compared to the typical appearance for this species.

On review of clinical pathology subsequent to the examination, aspartate aminotransferase (AST) was noted to be increased 3-fold from historic baselines for this individual which suggested an unidentified hepatic insult (Table 1). Infectious disease consultation suggested a probable viral cause due to local community presentations in similarly aged human males with parainfluenza virus that presented with a consistent non-specific presentation and AST rise. Additionally, clinical pathology was not reflective of a bacterial hepatitis as hemogram presented a low normal, lymphopenic white blood cell count. Two voided urine samples analyzed prior to this procedure and catheterized urine sample obtained during the procedure were concentrated as consistent with dehydration prior to the examination, and then within normal range with rehydration during the procedure. All cellular profiles were consistent with mucosal voiding or catheterization without pyuria, hematuria, or more than trace proteinuria.

**Table 1:** Hepatic function serum chemistry parameters from gorilla SSP individuals over 10 years of age, assessed by signalment, and health status and separate presentation of presumed ACE-I hepatotoxicity case.

	**ALT (U/L)**	**tbil (mg/dl)**	**ALP (U/L)**	**AST (U/L)**	**GGT (U/L)**	**LDH (U/L)**
Male ≥ 10 year^a^	40.2 ± 28.4	0.68 ± 0.52	265 ± 527	39.6 ± 27.9	42.3 ± 98.4	557 ± 316
Live 10–25 year^b^	27.5 ± 15.7	0.5 ± 0.23	436 ± 224	25 ± 12.2	31.3 ± 62.3	420 ± 239
Healthy^c^	35.5 ± 18.2	0.56 ± 0.50	272 ± 455	33 ± 23.5	53.2 ± 93.6	523 ± 381
Ill^d^	41.5 ± 50.5	0.66 ± 0.60	265 ± 291	54.1 ± 198.3	81.2 ± 145.4	592 ± 395
Non-ACE^e^	37 ± 31.3	0.57 ± 0.49	279 ± 435	39.9 ± 127.1	64.4 ± 114.5	541 ± 400
ACE^f^	39.4 ± 39.1	0.7 ± 0.73	221 ± 229	40.5 ± 36.6	54.3 ± 112.5	618 ± 296
Enalapril^g^	45.3 ± 63.4	0.84 ± 1.2	185 ± 67	40.1 ± 34.5	51.1 ± 49.2	600 ± 348
**CASE PRESENTATION**						
Baseline	37	0.7	215	37	33	402
Diagnostic^h^	72	1.2	145	95	33	534
Diagnostic^i^	125	1.3	222	287	76	1,995
Convalescent^j^	29	0.7	175	22	35	353

a *Living and deceased male gorillas of healthy or ill status (n = 189) with 466 panels assessed*.

b *All living 10–15 year old gorillas of both sexes and of healthy or ill status (n = 28; 13.15) with 65 panels assessed*.

c *All living and deceased gorillas of both sexes and healthy status (n = 343; 167.176) with 651 panels assessed*.

d *All living and deceased gorillas of both sexes and ill status (n = 193; 84.109) with 333 panels assessed*.

e *All living and deceased gorillas of both sexes and of healthy or ill status (n = 338;135.203) that were not prescribed ACE-I with 815 panels assessed*.

f *All living and deceased gorillas of both sexes and of healthy or ill status (n = 63; 55.8) that were prescribed ACE-I with 167 panels assessed*.

g *All living and deceased gorillas of both sexes and of healthy or ill status (n = 63; 18.2) that were prescribed enalapril with 52 panels assessed*.

h *Sampled at 7 days after presentation with presumed ACE-I hepatotoxicity*.

i *Sampled at 15 days after presentation with presumed ACE-I hepatotoxicity with ongoing enalapril administration*.

j *Sampled at 42 days after presentation with presumed ACE-I hepatotoxicity and after 28 days without receiving enalapril*.

*ALT, alanine aminotransferase, tbil, total bilirubin, ALP, alkaline phosphatase; AST, aspartate aminotransferase; GGT, gamma-glutamyltransferase; LDH, lactate dehydrogenase*.

Supportive care was focused on oral fluids and preferred diet item supplementation, but it was insufficient to maintain this individual. Over the following week, its health status declined precipitously to essentially complete anorexia, including refusal of enalapril which started on the 12th day of illness. Presenting weight (175 kg) was reduced by 10% over only 8 days. It became necessary to institute physical isolation of this individual from its peers, both for management and to prevent injury from them in its compromised state. A second diagnostic procedure was performed with repeated assessments from the first examination, including abdominal ultrasound (Table 1), and additionally computed tomography. The presence of jaundice on physical examination and elevation of multiple hepatic enzymes confirmed the previously suspected hepatopathy. Hemogram had resolved to a more appropriate differential distribution, but absolute white blood cell count remained in the low normal range.

No definitive cause of this presentation could be identified. Although serology for available parainfluenza viruses (1, 2, and 3 – Virus Reference Laboratory, DIA) was positive, this status was unchanged from historic assessment of this individual. Aerobic blood and rectal pathogen cultures were negative. Available serology for potential bacterial (tularemia—National Veterinary Services Laboratories, plate testing; *Leptospira bratislava, L*. *canicola, L*. *grippotyphosa, L*. *hardjo, L*. *icterohemorrhagicae*, and *L*. *pomona*—Michigan State University Veterinary Diagnostic Laboratory, MAT) and viral (Hepatitis A total antibody—EIA; Hepatitis A IgM antibody, Hepatitis B surface antibody and antigen, and Hepatitis C Antibody—CMIA—Virus Reference Laboratory) etiologies consistent with hepatic disease were negative.

The gorilla remained anorexic and non-compliant with oral medication and feeding. Although no specific indication existed, presumptive antibiotic coverage (enrofloxacin, Bayer HealthCare, LLC, Shawnee Mission, Kansas, 500 mg IM, SID, by dart) was initiated, but administration success decreased over 5 days. Finally, at 9 days after the last dose of enalapril, the gorilla consumed a very small quantity of soft food.

Literature review revealed reports of hepatotoxicity attributed to angiotensin-converting enzyme inhibitors (ACE-I), such as enalapril (1–7). Hepatoprotection (Denosyl, Nutramax Veterinary Sciences Laboratories Inc., 1,700 mg PO SID to divided QID) therefore was initiated, and enalapril was permanently discontinued. Over the following 2–3 weeks, the gorilla returned entirely to normal health and appetite, and regained lost weight. The gorilla was anesthetized as previously described for a convalescent examination and laparoscopic hepatic biopsy 42 days after presentation. The biopsy demonstrated a neutrophilic hepatitis on histopathology. At 50 days post-presentation, the gorilla was returned to its troop.

## Materials and Methods

This manuscript is a clinical case report that was managed with the principles of good standard of care for zoological medicine within the US for a facility approved by the American Association of Zoos and Aquarium. Lincoln Park Zoo's internal management was fully apprised for the details of the treatment and the resolution of this case. However, the subsequent context to the gorilla population was endorsed and supported by the AZA's Species Survival Plan (*Gorilla gorilla*) and the internal research committee of Lincoln Park Zoo was apprised of this investigation, but per internal guidelines for the institution, as a case, it does not require review or IACUC processing.

### Species Context of ACE-inhibitor Hepatotoxicity

As a result of this case presentation, a solicitation was made to the 49 gorilla Species Survival Plan (SSP) holding institutions of the Association of Zoos and Aquariums (AZA) in collaboration with the Great Ape Heart Project based at Zoo Atlanta. The studbook was queried for all gorillas living and deceased that were over the age of 10 years. The signalment and details of ACE-I prescriptions were recorded for each gorilla. The most recent available serum chemistry panels were obtained, up to a maximum of three panels per gorilla, and health status at the time of sampling was recorded. For deceased gorillas, the cause of death and presence of hepatic pathology on postmortem examination also were recorded.

## Results

From the 423 individuals reviewed, living (*n* = 277; 131.146) and deceased (*n* = 146; 79.67) were roughly evenly divided between sexes in each category as listed first as males by number then females separated by period as punctuation. Only 15.3% (*n* = 65) of this entire population had been prescribed an ACE-I, with only one individual prescribed two different ACE-inhibitors (Table 2). Lisinopril was the most frequently prescribed ACE-I, administered to 37.3 individuals (*n* = 40). Enalapril had been prescribed at a dose as high as 50 mg PO BID. The youngest female to have been prescribed either enalapril or lisinopril was 37 years old, while the youngest male was 12 years for lisinopril and 10.6 years for enalapril. Duration of prescriptions varied: <1 year (*n* = 16); 1–5 years (*n* = 12); 5–10 years (*n* = 24); and >10 years (11).

**Table 2:** Summary of ACE-I prescribed in the SSP gorilla population over 10 years of age.

	**Enalapril**	**Lisinopril**	**Rampiril**	**Captopril**
Males (alive)	7^a^	29^b^	0	0
Males (deceased)	12	8	0	1
Females (alive)	0	2	1	0
Females (deceased)	2	1	2	0

a *This group included the male in this case presentation*.

b *This group included one male that had also been prescribed captopril*.

Only the hepatic function parameters were evaluated from the available serum chemistry panels, including alanine aminotransferase (ALT), total bilirubin, alkaline phosphatase (ALP), AST, gamma-glutamyltransferase (GGT), and lactate dehydrogenase (LDH). Average and standard deviations for these values were considered in a variety of categories as context for the case presentation (Table 1).

Only 4.8% (*n* = 7; 2.5) of the mortalities were attributable to hepatic disease as a primary cause of death; none of these individuals had been prescribed ACE-I. Twenty gorillas (13.7%) that died had been prescribed either enalapril (11.2) or lisinopril (6.1), although none of these individuals had hepatic histopathology consistent with ACE-I hepatotoxicity, and none had been presented for hepatic disease at the time of their death or euthanasia. It was concluded that the current case was the sole individual which had presented to date with probable ACE-I hepatotoxicity.

## Discussion

### Background of ACE-inhibitors

Angiotensin-converting enzyme inhibitors (ACE-I) are prescribed for congestive heart failure and associated hypertension. They function by several coordinated actions to modulate cardiac function by the reduction of cardiac work (4) Prevention of the conversion of angiotensin I to angiotensin II reduces smooth muscle contraction of blood vessels. Reduction of aldosterone synthesis increases sodium loss and reduces volume overload. Potentiation of bradykinins leads to vasodilation. This drug class entered the medical prescription armament with captopril in 1980. By 1985, cases of suspected hepatotoxicity were reported worldwide (4, 8). Reports continued even for chemically modified versions of ACE-I, such as enalapril and lisinopril that lacked the sulfhydryl group that was thought the source of toxicity (4, 5).

The frequency of hepatotoxicity for ACE-I is rare. It likely was masked in initial clinical trials as a similar frequency of hepatic effects were reported in the placebo groups. Typically, concurrent hepatotoxicity is identified during the 1–2 years following release of a drug when sufficient persons have consumed the product to identify the issue (9–11). As of 2010, it was reported that ~159 million prescriptions were written for ACE-I in the US each year since 1989, and enalapril has been one of the top 12 medications of any drug class prescribed annually (12). With incidence of liver disease at 1:10,000–1:100,000, such as with ACE-I, a prospective study would require 30,000 subjects to detect a single case with 95% confidence (9–11). For perspective, most pharmaceutical trials are conducted with fewer than 2,000 human subjects, and even that number is 5-fold the US managed gorilla population both historically and currently over the age of 10 years as consistent with the presented individual. The total number of ACE-I prescriptions for gorillas is only 65 for this same group (Table 2).

Generally, adverse drug reactions (ADR) from pharmaceuticals can be related to the drug's mechanism of action, cumulative dose, or duration of administration (13). However, ACE-I have an unpredictable pattern of ADR unrelated to any of these factors. The mechanism is suspected to be an immunologic, although not an allergic response to the chemical structure of the drug as it goes through its metabolic process (5, 10, 11). Duration from initiation to ADR in humans has been variable from days to several years of administration (1, 2, 4–6). Recurrence of hepatotoxicity has been demonstrated for the same and different ACE-I for patients unfortunate enough to have been resumed on the products, so they are considered cross-reactive and very likely use a similar mechanism (3, 4, 7, 8).

Hepatotoxic ADR for ACE-I is most commonly cholestatic—measured by biliary compounds of ALP, bilirubin, or GGT. However, hepatocellular compromise—measured by functional enzyme increases of AST, or ALT, or a mixture of cholestatic and hepatocellular pathologies also have been reported (9). Elevations of 2–5 fold from baseline measurement of ALT or AST, with or without elevations of ALP, are considered consistent with hepatic damage (9, 10, 14, 15). In the gorilla of this case, ALT was 3.4-fold and AST was 7.8-fold above its historic baseline measurements, while ALP was largely unchanged. Even in severe human ACE-I toxicity, discontinuation of the prescription generally resulted in complete resolution of the clinical signs within 15 days (14), and full recovery over a few weeks (1, 10). Supportive care is the focus during the healing period as no antidote or reversal agent is available.

Since this drug class is hepatically metabolized using cytochrome P450 enzymes, other medications or chemicals which affect this metabolic pathway could increase the risk of ADR (2, 3, 16). Mulberry, as was consumed by this gorilla's troop the day prior to its presentation, has remarkable P450 (CYP34A) inhibition which is exceeded only by grapefruit chemicals (16–18). Although some studies have shown pre-treatment with cytochrome P450 inhibitors to reduce liver injury from ACE-I (12), the same studies have shown ACE-I cause liver injury. Different types of cytochrome P450 enzyme systems exist, and it is unclear how directly *in vitro* testing translates into *in vivo* expression (16); it is also unclear if an increase in serum ACE-I concentration can induce appearance of an ADR.

As an ADR, drug induced liver injury is suspected to cause from 10 to 50% of cases of acute hepatic failure, of which 13% are idiosyncratic injury triggered by non-acetaminophen drugs (9). Severe ADR occur with cardiovascular medications 2.4 times more often than with other medications and in most situations, clinical signs are unrelated to the cardiovascular system (13). Signs are vague in that while they are liver specific, no characteristic timeline or point of origin exists, so these ADR require both clinician awareness and diagnosis by exclusion of the more common causes of liver disease (14). A front line tool was developed for medical professionals to rate such presentations and critically assess ADR potential (15). On this scale, and by eliminations of viral hepatitis and human-specific risks such as travel, alcohol abuse, or blood transfusions; with consideration of time of onset, hepatic enzyme changes noted, and their return to normal range after discontinuation of enalapril; and known hepatotoxicity of ACE-I, the gorilla of this case was assigned to the category of “probable” hepatotoxicity from enalapril (15, 19).

Pharmacovigilance begins with awareness of potential ADR as documented for a given drug. However, advance knowledge for an SSP species is considered highly improbable simply due to the small number of individuals. It also must be considered that nearly every pharmaceutical used in zoos is done so in an extra-label manner, usually prescribed without any clinical trial in the patient species. Even in the approved species, any drug has the ability to produce an ADR (19). These details should be reported to organized species or taxon groups to minimize the potential for future concerns and reduced interval to action in future presentations. Humans, though related to gorillas taxonomically, are not obligate folivores. Frequently, plants contain multiple chemicals with unknown metabolic impact, and herbivores routinely ingest many more of these than a human ever will, and in portions beyond that consumed by a human (16, 17). Managers are asked to consider that any medication can result in an ADR. A sudden change in health status during even a chronic, and generally safe, prescription is an occasion to call the medication itself into question. Thorough history of consumed enrichment or browse must be part of the history to enhance awareness for this possibility and allows for more prompt withdrawal of the offending product.

## Data Availability Statement

The datasets generated for this study are available on request to the corresponding author.

## Author Contributions

KCG, JL, and KAG contributed to the clinical management and resolution of this case, subsequent manuscript revision and completion, and agree to be accountable for the content of the work. KCG completed assessment of background data and individual context.

### Conflict of Interest

The authors declare that the research was conducted in the absence of any commercial or financial relationships that could be construed as a potential conflict of interest.
